# Book review

**DOI:** 10.3402/ijch.v74.27032

**Published:** 2015-02-17

**Authors:** Fikre Germa

**Affiliations:** Brantford General Hospital, Brantford, Ontario, Canada, N3R 1G9

Review of *Circumpolar Health Atlas*, edited by Kue Young, Rajiv Rawat, Winfried Dallmann, Susan Chatwood, Peter Bjerregaard (2012). Toronto, Canada: University of Toronto Press. 198 pp. ISBN 9781442644564.

If only I had the *Circumpolar Health Atlas* when I started working in Canada's northern communities some years ago, it would have given me a valuable overview of the many factors that ultimately affect clinical care in the north as well as insights into the complexities of the region. I could have done better health promotion and advocacy in helping patients understand and adapt to change.

Although the title of this resource refers to health, the dimensions discussed range from geography to history and from genetics to health care systems and social determinants critical to the well-being of the population. This informative atlas is beautifully illustrated with graphs, maps, charts, tables and photographs. The book also has a good bibliography.

The atlas is organized in five parts. Part One is an overview of the geography; Part Two covers cultures and languages; Part Three looks at diseases and health outcomes; Part Four looks at the distribution of different types of health determinants; and Part Five describes health care system policies, resources, education and research. The information is presented pictorially and in well-written summaries, making the material digestible, useful and enjoyable.

I was pleasantly surprised at the many things I learned, even though I had worked in the north. For a start, I had believed the Arctic geographically was mainly Canadian, American and Russian but discovered it includes other countries such as Greenland, Iceland, Sweden, Norway and Finland. The book describes this enormous region's socio-economic and political importance and how it is being affected by climatic change and natural resources development.

I also gained a further appreciation of the cultures of the Inuit, Dene, Norse and others who populate this region, and learned about the existence of the Dorset culture, which was replaced by the Thule culture, the direct ancestors of the Inuit. This area has been colonized, decolonized and the culture affected by migrations of various peoples. The book reinforced my conviction that culture matters.

In terms of disease patterns, I was struck to learn that outbreaks of botulism occur in the Inuit region in the north. This happens partly because of the traditional processing of meat and fish, which involves fermentation for anaerobic storage at low temperatures without salting. Zoonotic infections are prevalent because of the hunting practices, and it is feared that climatic changes could further influence this.

The book has value for health practitioners outside the Arctic. They could, for example, learn more about how diet change and genes have interacted to cause diabetes and heart disease. Health care workers in larger centres would find the *Circumpolar Health Atlas* a valuable resource for a better understanding of northern patients transferred to them.

The charts show trends such as suicide rates in selected indigenous populations. Another chart shows tuberculosis incidence among natives of Greenland and Alaska and the Canadian Inuit compared to Canada since the 1950s, and the substantial decline of TB, thanks to public health interventions. Other charts show the emergence of cancer and other chronic diseases. The Inuit have the highest incidence rate of lung cancer, a pattern that was news to me.

The section on health care systems compares how health care systems are organized and financed in circumpolar regions. The material is helpful for identifying best practices, solutions to common problems, and opportunities for collaboration.

The book also looks forward with discussions about rapidly emerging opportunities for research and alliances. I believe the atlas will help move forward collaboration with southern partners.

The authors, who are from Denmark, Greenland, Norway and Canada, are well known, and the editors are distinguished scholars who know the region well. This makes the book authentic.

As the authors say, there is something for everybody in the *Circumpolar Health Atlas* – for those in health care and the general reader, and for those who work in the north and those who know nothing about it. This remarkable and beautiful book provides an amazing introduction to the Arctic, its nature and the health care system. I recommend it whole-heartedly.

*Fikre Germa, MD* Brantford General Hospital Brantford, Ontario Canada, N3R 1G9

